# Exploring the feasibility of ex-post harmonisation of religiosity items from the European Social Survey and the European Values Study

**DOI:** 10.1186/s42409-022-00038-x

**Published:** 2022-09-30

**Authors:** Eva Aizpurua, Rory Fitzgerald, Julia Furtado de Barros, Gregorio Giacomin, Vera Lomazzi, Ruud Luijkx, Angelica Maineri, Daniela Negoita

**Affiliations:** 1 European Social Survey ERIC, City, University of London, London, UK; 2 Department of Sociology, Tilburg University, Tilburg, Netherlands; 3 Department of Sociology and Social Research, University of Trento, Trento, Italy; 4 Department of Human and Social Science, University of Bergamo, Bergamo, Italy

**Keywords:** Survey methodology, Cross-national data, Ex-post harmonisation, Source and target variables, Validity

## Abstract

This paper examines the feasibility of ex-post harmonisation strategies using European Values Study (EVS) Wave 5 (2017–2020) and European Social Survey (ESS) Round 9 (2018–2019) data across 17 countries. The study shows an empirical assessment of the comparability of four items measuring religious behaviours (belonging to a religious denomination at present/in the past, religious services attendance, and praying), captured in both surveys. The novelty of this paper lies in the analytical comparison of religiosity indicators that are rarely assessed from a comparative perspective.

The harmonisation strategy was based upon several analytical techniques that seek to determine similarities and differences between the selected items in terms of (a) their validity, by examining their correlations with a set of sociodemographic and substantive correlates, (b) their distributions, supplemented by visual comparisons and relevant statistical tests, and (c) item non-substantive shares. The findings pointed to the most consistency among the partial correlations, where individual religiosity produced the most differences between the surveys. Distributions produced the most discrepancies that also corresponded to less similarity across variable categories as gauged by Duncan’s index. This paper is descriptive and exploratory in its aim. It can be taken as a jumping-off point for future research where the time series of these two surveys, and potentially others, can be examined across aggregate levels (e.g. birth cohorts, countries).

**Supplementary Information:**

The online version contains supplementary material available at 10.1186/s42409-022-00038-x.

## Introduction

The ex-post harmonisation of survey data from different sources is a blossoming field of research and consists of using existing data to build an integrated dataset in which the reliability and validity of the outcome measurements is preserved (Wolf et al., 2016). The methodological harmonisation procedures aim at obtaining compatible measurements of surveys over time, across countries, projects, or a combination of those (Dubrow & Tomescu-Dubrow, 2016; Wolf et al., 2016). In the survey research literature, harmonisation can occur at two different stages: (1) during the design of the survey (ex-ante harmonisation); (2) after data collection (ex-post harmonisation) (Wolf et al., 2016).

Pooling data from different data sources enhances possibilities of empirical investigations (Wolf et al., 2016). Recent examples of large-scale survey data ex-post harmonisation projects include the Survey Data Recycling project-SDR (Tomescu-Dubrow & Slomczynski, 2016) and the Church Attendance and Religious Change Pooled European (CARPE) dataset (Biolcati et al., 2020). The main methodological challenges reported in the literature point to variables that have different measurements or scales (especially scales in attitudinal questions), differences in classification schemes and/or numeric codes assigned to each category, or different question wordings, even when the underlying concept is the same (Tomescu-Dubrow & Slomczynski, 2014, 2016). However, this conceptual overlap across surveys is often assumed yet rarely tested empirically.

## The European Social Survey (ESS) and the European Values Study (EVS)

The European Social Survey (ESS) and the European Values Study (EVS) are large, cross-national social survey projects that collect data in most European countries. Despite many differences between the two, there are also important similarities. Combined, the two data sources offer a complementary and substantial perspective to analyse value change in Europe. The EVS and the ESS questionnaires cover a variety of domains relevant for social sciences with high methodological rigour. Although conceptual coverage is similar, the use of particular indicators secures the specificity and the identity of each survey.^1^ The set of questions in both questionnaires allows monitoring change and continuity of values and attitudes in the domains of family, gender roles, work, religion, immigration, politics, and society. Both surveys were traditionally carried out as face-to-face interviews to individuals selected with probability-based sampling designs. Both surveys target all residents in private households in a country, regardless of nationality and language. Challenges including increased costs and deteriorating response rates led the EVS to experimentally implement mixed-mode innovations in the 5th wave in six countries (Luijkx et al., 2021). Since 1981, the EVS has been conducted every 9 years (1990, 1999, 2008, 2017) whereas the ESS is a biannual survey which, since 2002, has fielded 10 rounds of data collection using a core module that is repeated over time, new and repeated rotating modules, as well as a comprehensive set of sociodemographic variables.^2^ Some differences regarding fieldwork duration and organisation as well as slightly distinct criteria to define the target population exist between the two surveys (ESS, 2018; EVS, 2020b). All in all, both data sources are widely used in the scientific community to inform policies, and as an important source of methodological innovation in comparative survey research.

## The current study

In the framework of the ESS-SUSTAIN-2^3^ project, the EVS and the ESS investigated different scenarios for a potential collaboration in data collection, one of the potential scenarios being a joint data collection and, consequently, the need to create an integrated dataset including data from both surveys. Since the original questionnaires were not designed to be comparable, this paper will attempt to assess the comparability of selected items and determine whether ex-post harmonisation is feasible. In this context, devising harmonisation strategies to pool the two data sources without compromising the long-standing time series is a key element.

This study presents the first stages of evaluation of such a harmonisation process, in which data collected by the EVS (Wave 5) and the ESS (Round 9)^4^ across 17 countries were used to examine the comparability and conceptual overlap of selected items. This paper presents the results of the methodological procedure implemented to (a) empirically investigate the conceptual overlap between matched items of ESS and EVS questionnaires in terms of validity of measurement, and (b) compare the distributions to understand differences in the actual measurement.

To illustrate the proposed methodological operationalisation, we focus on four item pairs tapping on religiosity, including (1) belonging to a religious denomination, (2) ever belonged to a religious denomination, (3) attendance at religious services, and (4) praying frequency. The criteria used to select items to be compared were based on similarity in question wording and attributes, interviewer role, answer categories, and showcards layout.^5^ Moreover, the conceptual construct represented by these items covering distinct dimensions of religiosity informs the ongoing debate on the topic (Biolcati et al., 2020; Voas, 2009).

Previous experiences such as the SDR and the CARPE projects have shown that, although strategies to accomplish ex-post harmonisation differ widely, a common preoccupation lies in handling the methodological variation to guarantee high quality target variables (Biolcati et al., 2020; Tomescu-Dubrow & Slomczynski, 2016). The SDR project brings together information on equivalent measurement of political behaviour, social attitudes, and demographics from 22 international surveys worldwide, including 89 waves over 50 years (Tomescu-Dubrow & Slomczynski, 2014, 2016). The CARPE aggregated dataset includes a harmonised, country-level aggregate measure of church attendance across all available rounds of 5 data sources (ESS, EVS, Eurobarometer, International Social Survey Programme-ISSP, and World Values Survey-WVS) spanning over 45 European countries (Biolcati et al., 2020). Compared to these projects, our contribution consists of a shorter time span and a smaller number of surveys/waves including the latest available round of ESS and EVS data. However, compared to CARPE, we expand the comparison to multiple dimensions of religiosity, and focus on pooled data at the individual level. Our study also complements SDR by assessing the conceptual overlap of items prior to carrying out the ex-post harmonisation procedures. The relevance of the proposed empirical comparison is not only to shed light for future data harmonisation and cooperation between the two surveys but also (a) to provide information for researchers to understand whether survey data from different sources measure the same concepts and can confidently be pooled for analytical purposes and (b) for survey methodologists, to get insights into how differences in wording and methodology affect measurement and comparability.

In the following sections, we describe the criteria used to select the items and countries to be compared and the analytical plan. This effort purports to assess the feasibility of ex-post harmonisation and identify potential issues while exploring solutions during the process to ultimately offer insights for researchers interested in data harmonisation and its applicability.

## Methods

### Data and measures

In an initial phase of the project, members of both teams compared the items in English of each questionnaire and assessed their similarities based on 17 attributes clustered in four domains as shown in Table 2 in Appendix 1. These criteria were adapted from the Survey Quality Predictor (SQP), an online tool designed for predicting survey quality by using the information on the questions’ features (Saris & Gallhofer, 2014). The more shared attributes, the more compatible the pairs of items were considered. As a result, current and past belonging items achieved a consistency of 100% (indicating that all 17 attributes coincided in the EVS and ESS), followed by attendance at religious services (94%) and praying frequency (88%). Despite very similar question wording and response scales, religious services attendance differed on the label qualifiers (Table 4 in Appendix 1). The question wording for frequency of praying was very similar in both questionnaires and both response scales coincided, with the exception of one category (#5 which in ESS read as “Only on special holy days”, whereas in EVS “Several times a year” (see Table 5 in Appendix 1)). An overview of the source variables wording and measurement scales as well as the target items is provided in Tables 3, 4 and 5 in Appendix 1.

Regarding the correlates used to the compare the validity of the measurements, their selection was based on theoretical grounds suggesting that aspects of religiosity are different depending on sociodemographic characteristics such as age, sex, educational level, and income (Lemos et al., 2019; Schwadel, 2011, 2015). Notwithstanding variation in religiosity patterns across Europe, extant research has often pointed out some common trends. For instance, older people are more likely to attend church and to be more religious than younger individuals (Halman & Draulans, 2006; Vezzoni & Biolcati-Rinaldi, 2015; Voas, 2009). Substantial sex differences in religious practices and beliefs have also been documented in previous studies. Women tend to be more religious and to participate more in religious activities when compared to men due to cultural and socialisation mechanisms (Halman & Draulans, 2006). Higher education is likely to be negatively associated with church attendance and religious affiliation (Ruiter & van Tubergen, 2009). In addition, people who are not in paid employment are expected to be more religious than employed individuals, since those who are not working have more time available and fewer sources of identity and inclusion (Halman & Draulans, 2006). In turn, religious identity, the indicators of religious practice and religious belonging all correspond to empirically observable behaviours underlying the multidimensional concept of religiosity and therefore are frequently found to be strongly interconnected (Molteni & Biolcati, 2018).

The distributions of each correlate, disaggregated by survey and country, are displayed in Table 6 in Appendix 1. In order to achieve meaningful comparisons between surveys, the correlates were harmonised to maximise their similarities in terms of measurement and conceptual substantivity as shown in Table 7 in Appendix 1. As for the employment status variable, a target variable was generated which discerns between paid work (full time, part-time employees, and self-employed) and not in paid work (all the other categories). With regard to education, the source variables were recoded into a new indicator clustering the respondents based on their achieved level of education. The larger difference pertains to individual religiosity where the source variable for the EVS (3-point, nominal scale), with three categories, was reduced to two categories distinguishing those who identify themselves as religious from those who do not (not religious and atheist), to better mirror the ESS question tapping on the degree of religiosity (see Table 7 in Appendix 1).

### Analytical approaches

Prior to the analysis, a joint dataset was created by combining a selection of items from the EVS 2017 Integrated dataset (2020b) and the ESS Integrated file Round 9 dataset (2018). A few steps were undertaken, as shown in Table 8 in Appendix 1, to increase the comparability of the data.

To examine the comparability of the items between the surveys, several analytical strategies were carried out. First, we examined the validity of the items by means of Spearman’s rank-order correlations, accompanied by 95% confidence intervals (CIs) obtained by bootstrapping (1000 repetitions), with a set of sociodemographic characteristics: age, sex, education, paid work, and individual religiosity. Spearman’s correlations were chosen given the ordinal nature of most of the variables. Partial correlations were used instead of regular bivariate associations to control for the impact of the other correlates. Consistency in direction and magnitude of the coefficients across the two surveys along with overlapping CIs will be considered indication of similar validity between the two surveys and therefore comparable in terms of conceptual overlap.

Second, to detect substantive differences in the measurements, the distributions of the items were compared visually (histograms) and using relevant statistical tests depending on the nature—ordinal or binary—of the variable (Mann-Whitney *U* test and chi-square tests). The Duncan Dissimilarity Index was used to quantify the size of the differences in the measurement, as this measure can be interpreted as the proportion of observations that would need to change categories of a certain item in one survey to have the same distribution as in the other survey. In line with previous research (Biemer et al., 2018), the threshold in this paper to consider measurements similar is at least .10.

In addition to the comparison of the distributions of substantive values non-substantive responses were compared for each pair of items, assessing the extent to which the proportion of non-substantive values is comparable between the two surveys for each of the countries (for this, *z*-tests were used). The amount of non-substantive answers (e.g. refusals, do not know responses—see Table 8 in Appendix 1 for the full list) has been previously used as an indicator of data quality (Aizpurua et al., 2018; Cernat & Revilla, 2020). In the other univariate and bivariate analyses, these non-substantive values were handled by using a listwise deletion approach which led to drop between 2.5% and 4% of the observations from the analyses, depending on the target variable and country.

All the analyses were conducted using weighted data.^6^ Because of the differences in available weights for the two surveys, weighted analysis will be based on the best available weights^7^ for within-country analysis in each case.

Along with the variability stemming from the EVS and the ESS methodology, the country-specific operations introduce additional variation around the sampling frames, sample sizes, and response rates. Therefore, analyses were performed separately for each country. To aid the interpretation, countries were ranked based on their similarity, looking at the following cumulative set of criteria: (1) fieldwork conducted less than 1 year apart, (2) analogous sampling strategy,^8^ (3) similar sample sizes, and (4) similar response rates. Only three countries satisfied all four criteria (Germany, Norway, and Slovenia). Once we loosened the resemblance of the response rates, three additional countries were added (Estonia, Poland, and Sweden). Similarly, when the sample size affinity criterion was set aside, Finland, the Netherlands, and Switzerland were included. Finally, once the requirement of similar sampling design was removed, eight more countries were incorporated (Austria, Denmark, France, Great Britain, Hungary, Italy, Montenegro, and Serbia). Consistency in the outputs (Spearman’s correlations, distributions, Duncan’s test, and non-substantive responses) is expected to be stronger in countries with more similar methodologies. In countries with large differences in the ways data are collected (e.g. different sampling strategy), differences in methods may interact with differences in measurement, magnifying—or perhaps concealing—discrepancies.

## Results

### Currently belonging to a religious denomination

**Fig. 1:**
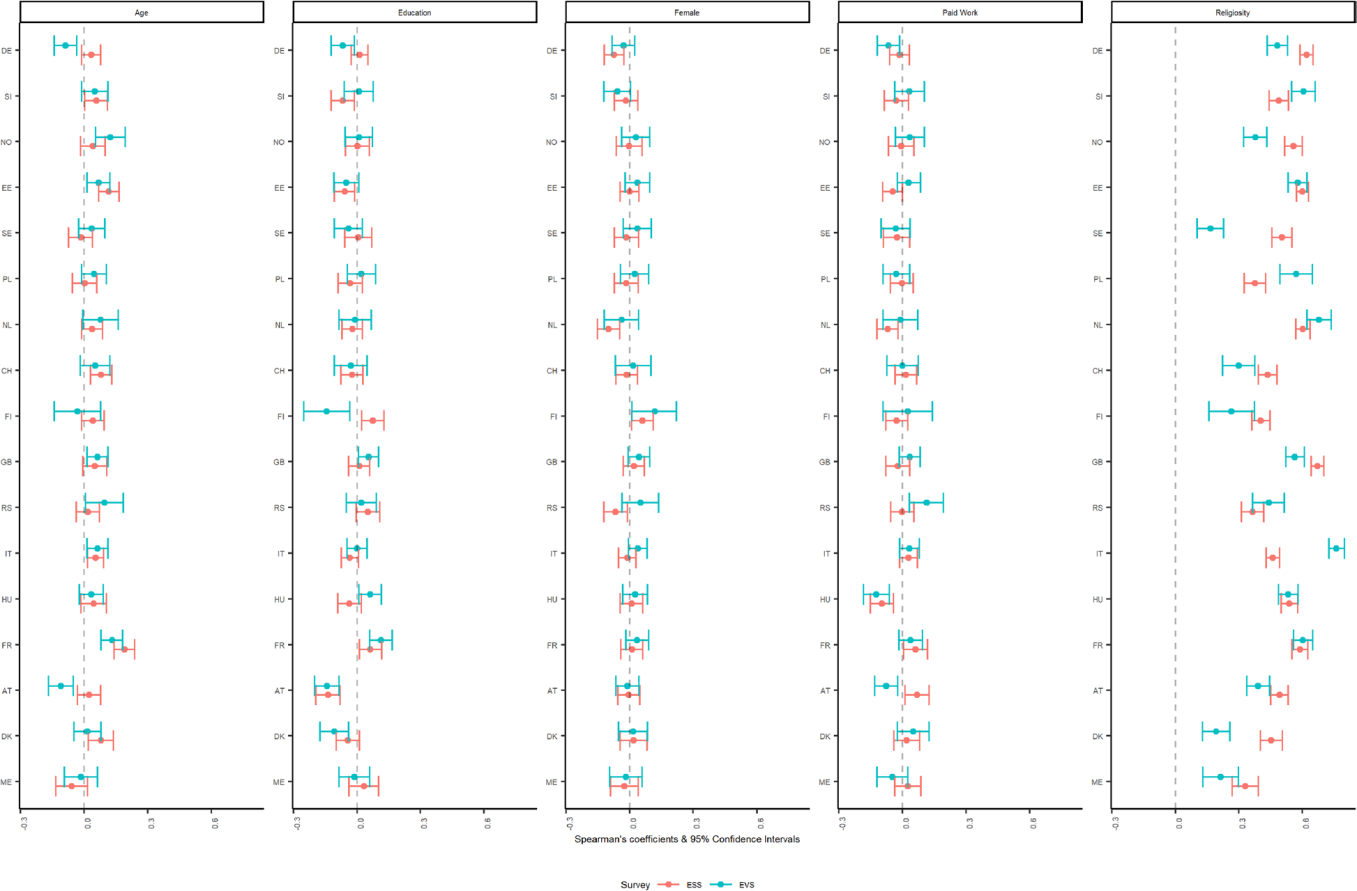
Spearman’s partial correlations of belonging to a religious denomination with selected sociodemographic and substantive items

Spearman’s correlations (values and 95% CIs) for currently belonging to a religious denomination (binary variable) with other correlates are displayed in Fig. 1. Overall, the coefficients are consistent in size and direction across the surveys. Age, education, sex, and employment status resulted in negligible and weak associations with belonging to a religious denomination. When looking at the CIs, the size and direction of the correlation with age in Germany and Austria is significantly different between the two surveys, with an indication of lower validity in the EVS due to a negative correlation which goes against the expectation (Halman & Draulans, 2006). Differences also occur for education in Finland and employment status in Austria, but in this case only the EVS displayed correlations in the expected direction (Ruiter & van Tubergen, 2009). As for individual religiosity, the correlations are for the most part positive as expected, and sizeable, however with varying strengths across countries and surveys. When looking at the CIs, most differences across the surveys were yielded for eight countries (Norway, Germany, Sweden, Poland, Switzerland, Italy, Great Britain, and Denmark), including countries with similar methodologies such as similar sample frame, sample sizes, and response rates.

The distributions of the EVS and ESS items measuring current belonging to a religious denomination are displayed in Figure 5 in Appendix 2. Overall, the EVS presented higher shares of affiliated people compared to the ESS. In both surveys, Poland registered the greatest percentage of adherents, whereas the country with the lowest percentage was Estonia. To test whether differences across the groups were significant, chi-square tests were computed. The differences in percentages of people adhering to a denomination were statistically significant in 15 of the 17 countries. Looking at the Duncan’s test, indicating the proportion of cases that would need to change categories to have the same distributions, we found that in most countries (9) said proportion was lower than .10, if not .05, indicating a small difference across surveys. Larger proportions, however, were found in Finland (*D* = .26), Sweden (*D* = .22), Denmark (*D* = .22), and Serbia (*D* = .21). As for the proportion of non-substantive responses, which can be found in Table 9 in Appendix 2, it was generally low in both surveys (< 3%). EVS—Italy exhibited the highest share (2.6%) against Poland in the ESS (2.0%). These differences were significant at the .05 level only for Estonia, Italy, Norway, and Poland.

### Ever belonged to a religious denomination

**Fig. 2:**
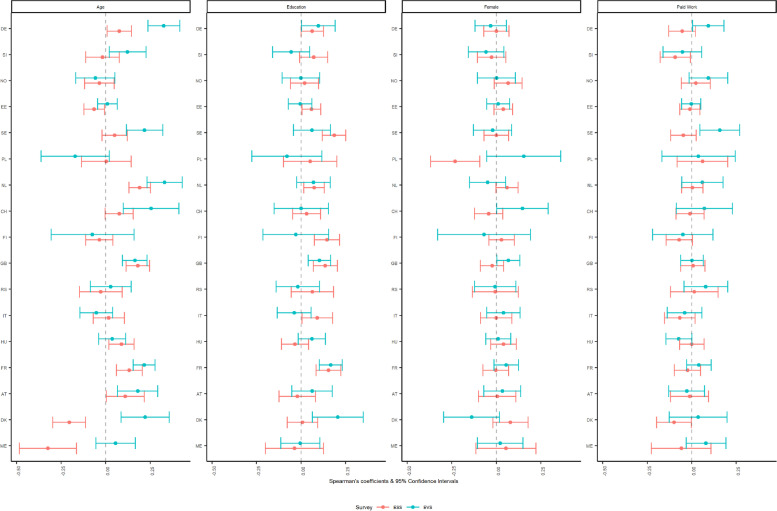
Spearman’s partial correlations of having ever belonged to a religious denomination with selected sociodemographic items (subgroup analysis)

Turning to past religious belonging (binary variable) in Fig. 2, correlations were estimated only for the sociodemographic characteristics, and this question was only administered to those who indicated not belonging to a religious denomination at present, making the correlation with current individual religiosity meaningless. Starting with age, the differences between the Spearman’s correlations across the surveys are minimal, exception made for three countries: Germany yielded non overlapping CIs and with the EVS relationship being stronger compared to the ESS, whereas Denmark and Montenegro showed non-overlapping CIs and reversed coefficients directionality, with positive associations in the EVS. Moving to education, sex, and employment status, the coefficients yielded overall negligible and small associations. The coefficients for sex in Poland and employment status in Sweden differ greatly as indicated by the non-overlapping CIs and opposite directions, with the EVS associations being positive. When compared to current belonging to a denomination, it is worth noting that the CIs of the correlations for past religious belonging are wider due to smaller sample sizes.

As for the distributions (Figure 6 in Appendix 2), countries in the EVS, by and large, displayed greater percentages of respondents responding positively when compared to the ESS, which is consistent with current religious denomination. Within the EVS, Finland presented the highest share of people that claimed to have belonged to a denomination, whilst within the ESS Austria was the country with the greatest percentage. Conversely, Serbia in the EVS and Hungary in the ESS had the lowest percentage of past affiliations that were around 5% for both countries. The chi-square tests showed that the surveys were significantly different, at .05 level, across all the countries, except for Estonia. When looking at the similarity between the surveys with the Duncan’s index, less than 10% of the respondents in 5 countries would have to change response categories to achieve equal distributions in the other survey. The rest of the countries yielded Duncan’s indices greater than .10, with Finland’s being the highest (*D* = .62) and Montenegro (*D* = .12) the lowest, indicating substantial differences across surveys. Regarding the non-substantive responses for having ever belonged to a religious denomination, shares were very low and differences mostly not significant (see Table 9 in Appendix 2), exceptions made for Poland, the Netherlands, Hungary, and Montenegro.

### Attendance at religious services

**Fig. 3:**
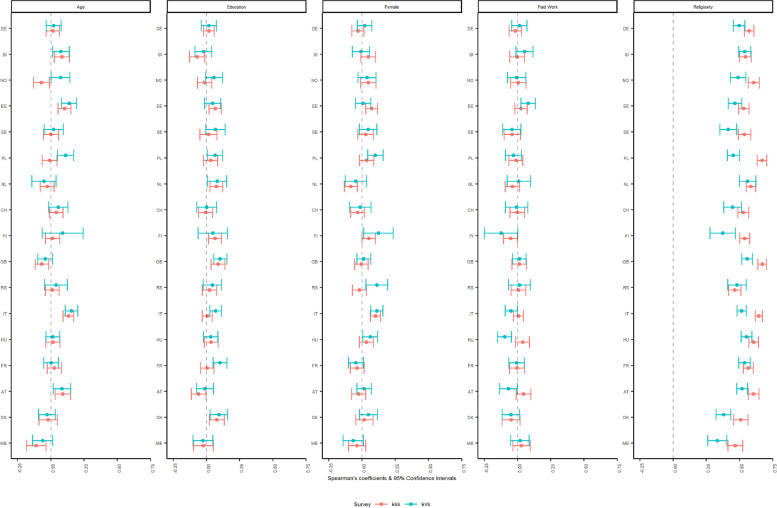
Spearman’s partial correlations of attendance to religious services with selected sociodemographic items

When looking at the correlation and associated 95% CIs for services attendance (ordinal variable, six categories) in Fig. 3, we see that the surveys did not deviate from each other when looking at age, education, and sex, showing negligible and small associations, which lends support to low yet comparable validity across the surveys. Moving to employment status, the coefficients for Hungary displayed opposite direction with non-overlapping CIs, with the ESS association crossing 0 and going against expectations (Halman & Draulans, 2006). When looking at individual religiosity, positive medium to strong correlations were registered across the groups indicating high validity, yet with differences across surveys with the ESS displaying significantly larger coefficients than the EVS in seven countries (Norway, Sweden, Poland, Finland, Italy, Great Britain, and Denmark).

The histogram in Figure 7 in Appendix 2 displays how, in both surveys, Poland produced the highest scores, while the lowest corresponded to Estonia in the EVS and the Netherlands in the ESS. In most of the countries the scores tended to pile up on the left side meaning that people tend to attend religious services from mostly never to only on holy days. The only exception to this trend is Poland that had skewed scores on the right-side indicating respondents that attend church once a week on average. The distributions were compared by means of a Mann-Whitney *U* test. When looking at Figure 7 in Appendix 2, distributions appear to be statistically different (*z* score significant at the .05 level) in 9 countries out of 17. In the countries with more comparable methodologies, distributions appear similar. The outcomes from the Duncan’s dissimilarity tests provided similarity across surveys in most of the countries (12), indicating that no more than 10% of the respondents would have to change response categories in the other survey. However, slightly larger coefficients were yielded for the other countries with Estonia (*D* = .15) being the highest and France (*D* = .11) the lowest. Regarding non-substantive answers, both the EVS and the ESS exhibited low percentage shares overall, with the exception of EVS-Serbia and ESS-Poland that had shares above 2%. The differences in non-substantive response across surveys were corroborated by statistical evidence only for Poland, Serbia, and Hungary as shown in Table 9 in Appendix 2.

### Praying frequency

**Fig. 4:**
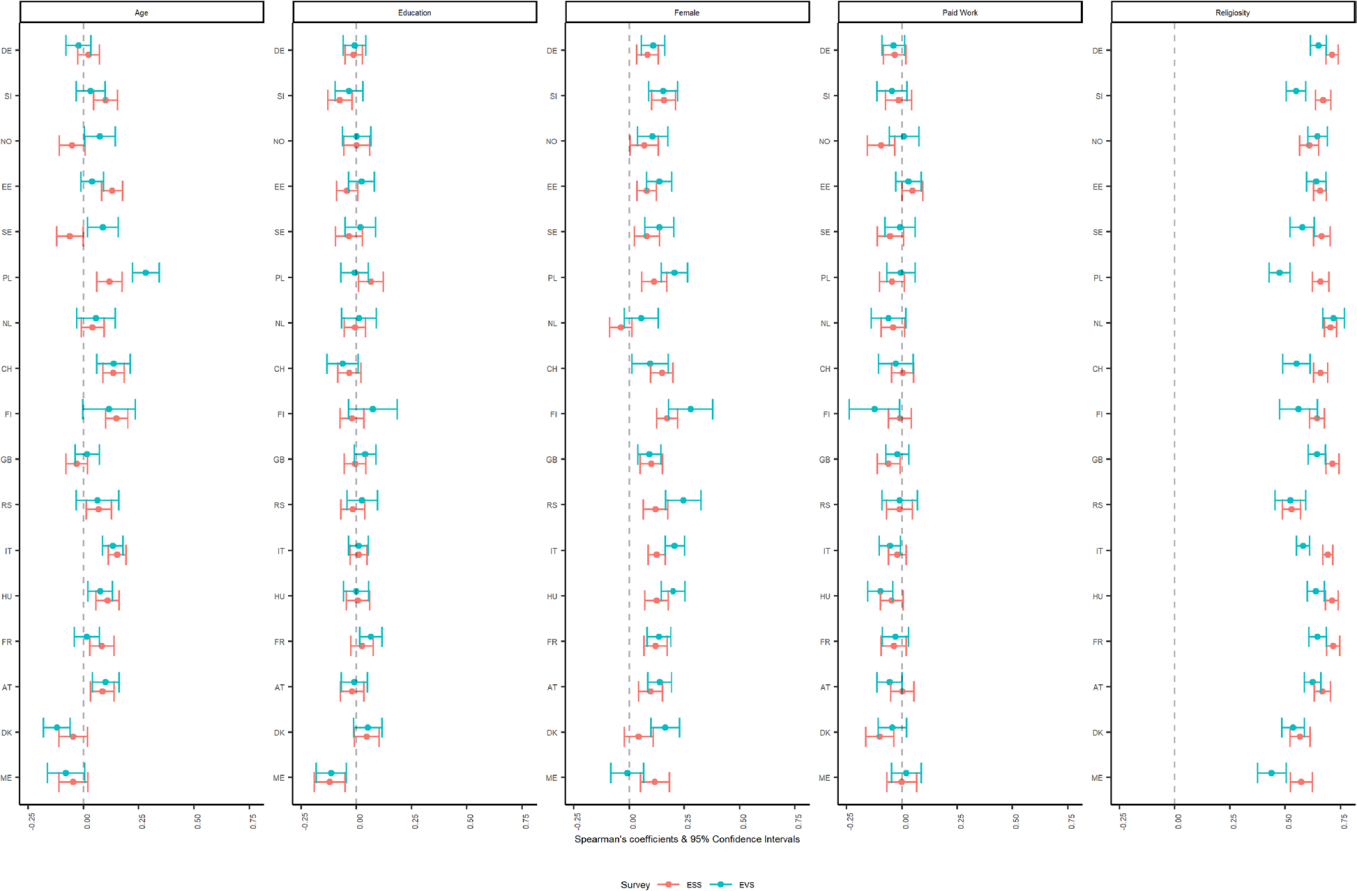
Spearman’s partial correlations of praying with selected sociodemographic and substantive items

**Fig. 5:**
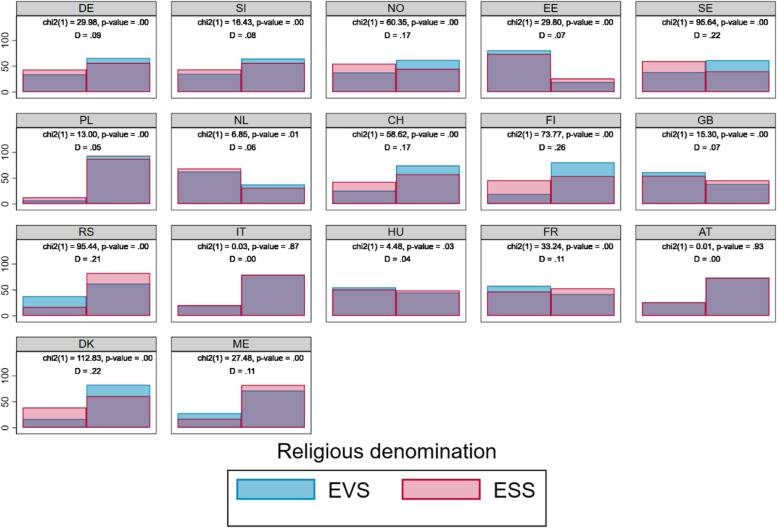
Bar charts for belonging to a religious denomination by country and survey

**Fig. 6:**
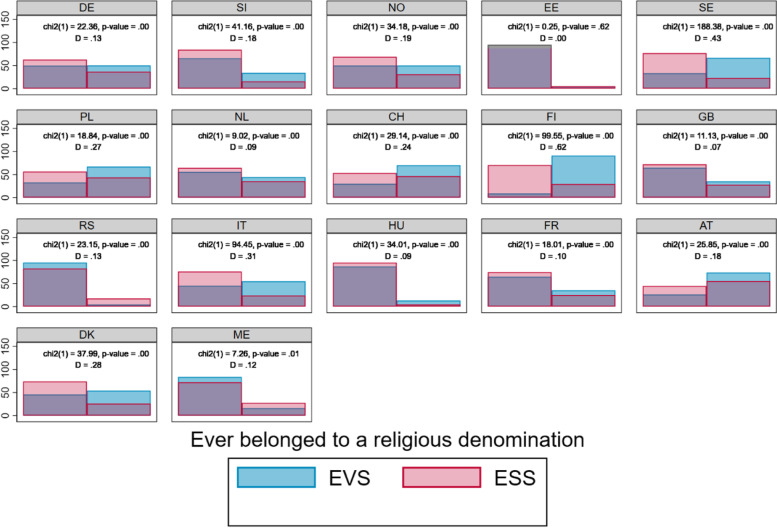
Bar charts for ever belonged to a religious denomination by country and survey

**Fig. 7:**
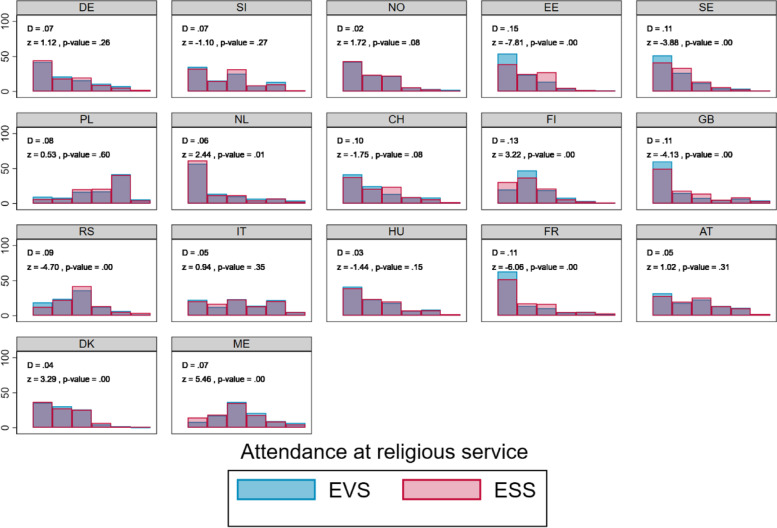
Histograms of attendance at religious services by country and survey

The results of the partial correlations for frequency of praying (ordinal variable, seven categories), disaggregated by survey and country, are displayed in Fig. 4 where all the correlations—apart from individual religiosity—showed negligible and weak strength indicating low validity overall. The findings showed most consistency for correlations with education, sex, and employment status as indicated by coefficients’ similar magnitude and direction along with overlapping CIs. The correlation values and 95% CIs with age yielded differences in Poland, with the EVS displaying stronger associations than the ESS, whereas in Sweden we see opposite direction and non-overlapping CIs, with the EVS showing positive associations. The associations with individual religiosity were once again stronger, indicating higher validity than with the sociodemographic variables, yet displayed differences in size between the surveys in Slovenia, Poland, Switzerland, Italy, and Montenegro, with the ESS displaying larger coefficients than the EVS.

**Fig. 8:**
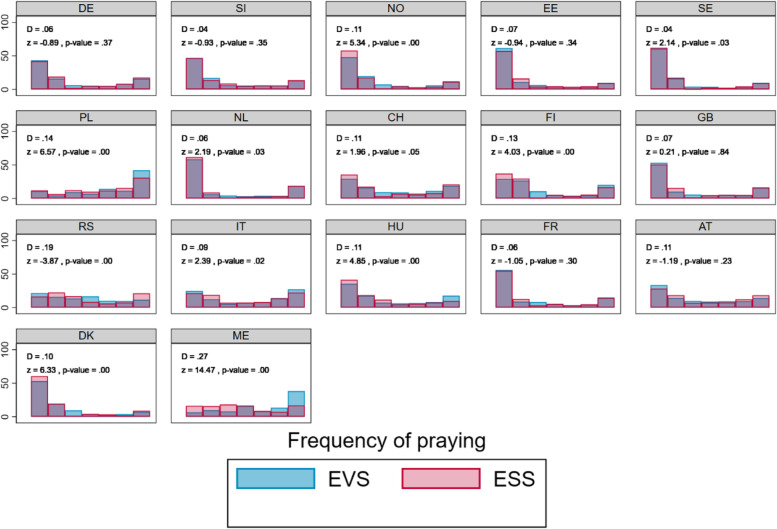
Histograms for praying frequency by country and survey

The distributions by survey and country in Figure 8 in Appendix 2 showed a tendency to select extreme answer categories on both sides, meaning that most respondents reported praying either never or every day.

Along with the visual inspection, a Mann-Whitney *U* test was carried out to determine whether there were differences in the distributions of praying frequency. The results showed that the surveys were significantly different (*z* score significant at .05 level) in 11 countries. In terms of Duncan’s Dissimilarity Index, 9 countries indicated high similarity between surveys with proportions lower than .10. The other countries yielded slightly larger indices as was the case for Norway (*D* = .11) and moderately larger such as Montenegro (*D* = .27). Overall, the non-substantive answers proportions ranged from very low (< 1%) to 3% for EVS in Montenegro and slightly above 8% for Poland in the ESS (Table 9 in Appendix 2). However, the differences between surveys were supported by statistical evidence only for Austria, Estonia, Hungary, Poland, and Montenegro.

### Summary of results

Our findings point to several conclusions (see Table 1). Spearman’s partial correlations with sociodemographic characteristics showed low validity but also, mostly, consistency between the surveys, especially for past religious belonging, followed by praying frequency, religious services attendance, and lastly, current religious belonging. Validity was higher when looking at the correlations with individual religiosity (substantive item), however that is also where most inconsistencies between surveys emerged. This might be due to discrepancies in the wording and scales of the source variables, with the EVS being a 3-category item dichotomised for the purposes of the analyses, whereas the ESS variable was measured on an 11-point scale. The differences yielded by the correlations with religiosity might, therefore, have been affected by the large differences in the scales of the source variables.

**Table 1:** Summary of the main results

	DE	SI	NO	EE	SE	PL	NL	CH	FI	GB	RS	IT	HU	FR	AT	DK	ME
**Current religious denomination**																	
*Inconsistencies in validity checks (partial correlations)*																	
Consistency	3/5	4/5	4/5	5/5	4/5	4/5	5/5	4/5	4/5	4/5	5/5	4/5	5/5	5/5	3/5	4/5	5/5
Light inconsistency (non-overlapping Cis)	1/5	1/5	1/5	0/5	1/5	1/5	0/5	1/5	0/5	1/5	0/5	1/5	0/5	0/5	0/5	1/5	0/5
Strong inconsistency (also different direction)	1/5	0/5	0/5	0/5	0/5	0/5	0/5	0/5	1/5	0/5	0/5	0/5	0/5	0/5	2/5	0/5	0/5
*Inconsistencies in distribution tests*																	
Chi-square significant at .05 level (df = 1)	Yes	Yes	Yes	Yes	Yes	Yes	Yes	Yes	Yes	Yes	Yes	No	Yes	Yes	No	Yes	Yes
Duncan’s index > .10	No	No	Yes	No	Yes	No	No	Yes	Yes	No	Yes	No	No	Yes	No	Yes	Yes
*Inconsistencies in proportion test of non-substantive responses*																	
*Z*-score significant at .05 level	No	No	Yes	Yes	No	Yes	No	No	No	No	No	Yes	No	No	No	No	No
**Past religious denomination**																	
*Inconsistencies in validity checks (partial correlations)*																	
Consistency	3/4	4/4	4/4	4/4	2/4	3/4	4/4	4/4	4/4	4/4	4/4	4/4	4/4	4/4	4/4	3/4	3/4
Light inconsistency (non-overlapping Cis)	1/4	0/4	0/4	0/4	1/4	0/4	0/4	0/4	0/4	0/4	0/4	0/4	0/4	0/4	0/4	0/4	0/4
Strong inconsistency (also different direction)	0/4	0/4	0/4	0/4	1/4	1/4	0/4	0/4	0/4	0/4	0/4	0/4	0/4	0/4	0/4	1/4	1/4
*Inconsistencies in distribution tests*																	
Chi-square significant at .05 level (df = 1)	Yes	Yes	Yes	Yes	Yes	Yes	Yes	Yes	Yes	Yes	Yes	No	Yes	Yes	No	Yes	Yes
Duncan’s index > .10	Yes	Yes	Yes	No	Yes	Yes	No	Yes	Yes	No	Yes	Yes	No	No	Yes	Yes	Yes
*Inconsistencies in proportion test of non-substantive responses*																	
*Z-*score significant at .05 level	No	No	No	No	No	Yes	No	No	NA	No	No	No	Yes	No	No	No	Yes
**Attendance at religious services**																	
*Inconsistencies in validity checks (partial correlations)*																	
Consistency	5/5	5/5	3/5	5/5	4/5	3/5	5/5	5/5	4/5	4/5	4/5	4/5	4/5	4/5	3/5	4/5	4/5
Light inconsistency (non-overlapping Cis)	0/5	0/5	1/5	0/5	1/5	2/5	0/5	0/5	1/5	1/5	1/5	1/5	0/5	1/5	1/5	1/5	1/5
Strong inconsistency (also different direction)	0/5	0/5	1/5	0/5	0/5	0/5	0/5	0/5	0/5	0/5	0/5	0/5	1/5	0/5	1/5	0/5	0/5
*Inconsistencies in distribution tests*																	
Mann-Whitney *U* test significant at .05 level	No	No	No	Yes	Yes	No	Yes	No	Yes	Yes	Yes	No	No	Yes	No	Yes	Yes
Duncan’s index > .10	No	No	No	Yes	Yes	No	No	No	Yes	Yes	No	No	No	Yes	No	No	No
*Inconsistencies in proportion test of non-substantive responses*																	
Z-score significant at .05 level	No	No	No	No	No	Yes	No	No	No	No	Yes	No	Yes	No	No	No	No
**Praying frequency**																	
*Inconsistencies in validity checks (partial correlations)*																	
Consistency	4/5	4/5	4/5	5/5	3/5	3/5	5/5	4/5	5/5	4/5	5/5	3/5	4/5	4/5	5/5	5/5	4/5
Light inconsistency (non-overlapping Cis)	1/5	1/5	0/5	0/5	1/5	2/5	0/5	1/5	0/5	1/5	0/5	2/5	1/5	1/5	0/5	0/5	1/5
Strong inconsistency (also different direction)	0/5	0/5	1/5	0/5	1/5	0/5	0/5	0/5	0/5	0/5	0/5	0/5	0/5	0/5	0/5	0/5	0/5
*Inconsistencies in distribution tests*																	
Mann-Whitney *U* test significant at .05 level	No	No	Yes	No	Yes	Yes	Yes	Yes	Yes	No	Yes	Yes	Yes	No	No	Yes	Yes
Duncan's index > .10	No	No	Yes	No	No	Yes	No	Yes	Yes	No	Yes	No	Yes	No	Yes	No	Yes
*Inconsistencies in proportion test of non-substantive responses*																	
*Z*-score significant at .05 level	No	No	No	Yes	No	Yes	No	No	No	No	No	No	Yes	No	Yes	No	Yes

*Source*: EVS 5(2020a), ESS 9(2018)–weighted

The distributions of the items appeared different between surveys, although when looking at the proportion of ‘misplaced’ cases, these were not overwhelmingly high. Indeed, high similarity across answer categories, as indicated by Duncan’s index, were obtained mainly for, in order, religious services attendance, current belonging, and praying. Non-substantive answers were overall low (as expected in interviewer-administered surveys), and differences were statistically significant only in a few countries. The outcomes for each analytical step for every variable were mixed, as was the case for past religious belonging that presented most inconsistencies for the distributions of the percentages of people having ever belonged to a religious denomination and less similarity between categories in terms of Duncan’s coefficients, whereas the partial correlations with the sociodemographic variables and the amount of non-substantive responses yielded the least inconsistencies.

Country patterns are mixed too. Countries like Austria, Denmark, France, Great Britain, Hungary, Italy, Serbia, and Montenegro, with more differences in the methodology displayed differences across surveys in at least one of the consistency checks. Countries with minimal methodological differences such as Slovenia, Germany, and Norway also presented a few inconsistencies (see Table 1) but to a lesser extent. Specifically, Germany, Slovenia, and Norway were the most similar in sampling frames, sample sizes, and response rates. They yielded least inconsistencies across the four selected items in the proportion of non-substantive responses, followed by the partial correlations, Duncan’s dissimilarity test, and finally differences in distributions (assessed by chi-square and Mann-Whitney tests at .05 level). The countries similar only in sampling frames and sample sizes—Estonia, Poland, and Sweden -showed least differences in the partial correlations, then equally inconsistent findings across the Duncan’s dissimilarity test and the proportion of non-substantive responses, with the worst performance in the distribution tests. Finland, Netherlands, and Switzerland were only similar in their sampling frames; they did not display any differences in the proportion of non-substantive response, whereas, starting from the least differences, they presented inconsistencies in the partial correlations, the Duncan’s dissimilarity test, and then the distribution tests. Finally, the countries that had the most variation across all the methodological benchmarks (Austria, Denmark, France, Great Britain, Hungary, Italy, Montenegro, Serbia), showed least inconsistencies in the partial correlations, followed by the differences in the non-substantive responses, the Duncan’s Dissimilarity test, and the distribution tests. In particular, distributions were more comparable across surveys in those countries than in countries with larger methodological discrepancies.

## Discussion

This paper examines similarities and differences between EVS and ESS items measuring the same concepts around religiosity. For this purpose, we selected four pairs of items that showed similarities in their attributes and potentially high conceptual overlap. Data from 17 European countries were included based upon several criteria: (1) data collection within 1 year, (2) similar sampling frames, (3) similar sample sizes, and (4) similar response rates. The countries were ranked starting from those that showed minimal variation to those that have cumulatively more differences around these benchmarks. Discrepancies in the measurement of religiosity items between the surveys were assessed on the basis of validity, frequency distributions and item non-response.

This study points to three main findings. First, the inconsistencies around the frequency distributions posit challenges for researchers interested in pooling data to provide estimates at the population level. This is in contrast with the results of Biolcati et al. (2020), who found evidence of consistency across survey programmes in building a harmonised measurement of church attendance. It is possible, however, that the harmonisation procedure implemented in the CARPE project, in which the ordinal variables were transformed into numeric probabilities of attending religious services at least weekly, successfully minimised measurement differences that we detected by looking at the full distributions.

Second, this paper showed overall comparable validity of the measurements across surveys, indicating a high degree of conceptual equivalence of the selected items. This finding suggests that researchers interested in correlational studies involving indicators of religiosity might confidently use a combination of the two data sources.

Third, discrepancies between surveys were larger in countries with larger differences in methodologies, as expected. While our study fails to isolate the contribution of each methodological discrepancy to the observed outcomes, it provides an indication that measurements are similar across surveys, net of methodological differences in data collection. To further explore these aspects and identify the sources of variation, large-scale experimental designs might prove fruitful.

Despite its contributions to the literature, this research presents a number of shortcomings. First, the analyses concerned a selection of variables with similar properties, limiting the possibilities to generalise to less homogenous variables and constructs. Second, the validity assessment was carried out with a limited number of correlates, and the relationships between our variables of interest and the correlates were generally low. For future research avenues, it would be helpful to expand the scope to additional variables, and to model the relationships in ways that better fit the data (e.g. by using interactions). Last, the analyses were carried out only on a single wave/round, not making full use of the longitudinal nature of the surveys. In addition to this, multiple comparisons were conducted, increasing the probability of identifying false positives (differences between the two surveys that are due to chance). We adopted this conservative approach, while also relying on effect sizes and ranges in our interpretation but recognise that differences might have been amplified by not correcting for multiple testing.

## Conclusions

Our outcomes lead to two main future implications for researchers interested in pooling the EVS and the ESS data. On the one hand, the inconsistencies around the frequency distributions posit challenges for researchers interested in estimates at the population level. On the other hand, this paper showed a high degree of conceptual equivalence of the selected items that provides ground for comparable results in terms of association among variables.

In general, the results presented in this paper are promising and show the potential to merge EVS and ESS data and their time series. The differences between different countries within the ESS and the EVS surveys are often bigger than differences within the same countries between the ESS and the EVS surveys. This paper is a contribution to the assessment of differences between countries in longitudinal surveys like the ESS and the EVS and yield the conclusion that we are on the right path. Further standardising and harmonising questions between international comparative surveys will increase future data quality.

### Supplementary Information

**Additional file 1: Table S1.** Comparison of EVS Wave 5 and ESS Round 10 items for belonging to a religious denomination.

**Additional file 2: Table S2.** Comparison of EVS Wave 5 and ESS Round 10 items for having belonged to a religious denomination.

**Additional file 3: Table S3.** Comparison of EVS Wave 5 and ESS Round 10 items for attendance at religious services.

**Additional file 4: Table S4.** Comparison of EVS Wave 5 and ESS Round 10 items for praying frequency.

## Data Availability

The datasets analysed during the current paper are available in (1) the European Social Survey repository available at 10.21338/NSD-ESS9-2018 and (2) the European Values Study repository available at 10.4232/1.13560. https://osf.io/r3swn/?view_only=cfd7a6496aae4925a26567fbcf8c3783.

## Appendix 1

### List of countries

Germany (DE), Slovenia (SI), Norway (NO), Estonia (EE), Sweden (SE), Poland (PL), Netherlands (NL), Switzerland (CH), Finland (FI), Great Britain (GB), Serbia (RS), Italy (IT), Hungary (HU), France (FR), Austria (AT), Denmark (DK), Montenegro (ME).

Tables 2, 3, 4, 5, 6, 7 and 8

**Table 2:** Criteria used to compare matched items during the attribute comparison

**Question attributes**	
Reference period	Time frame for which respondents are asked to report attitudes, behaviours or feelings
Reference period (detailed)	Specific frame for which respondents are asked to report attitudes, behaviours, or feelings (e.g. in the last 12 months, in your main job)
Balance of the request	Whether or not the question is formulated using both poles of the request
Part of battery	Whether or not the item belongs to a battery of questions (i.e. a set of statements measured using the same answer options)
Contingent on filter question	Whether or not the item is asked to a subset of respondents based on responses to one or more questions
**Interviewer role**	
Clarification	Whether or not the item contains clarifications, either within the question stem or as an instruction for the interviewer
Instructions	Whether or not the item includes specific instructions and, if so, for whom
**Response attributes**	
Type	Type of response
Number of categories	Total number of categories, excluding non-substantive responses (i.e., refusals, don’t know)
Range	Range of plausible values
Labels	The text linked to each response option
Label order	Ordering or direction of verbal labels
Polarity	Whether the scale is bipolar or unipolar
Neutral category	Whether or not there is a neutral midpoint
Symmetry	Whether or not the number of categories in bipolar scales is the same in both poles
**Showcards**	
Content	What showcard, if any, accompanies the survey question
Response layout	Layout of the showcard’s content

Criteria adapted from the Survey Quality Predictor (SQP) (Saris & Gallhofer, 2014)

**Table 3:** Religious denomination items

	ESS	EVS
**Belong to a religious denomination**		
**Question #**	C11	Q13
**Question stem**	Do you consider yourself as belonging to any particular religion or denomination?	Do you belong to a religious denomination?
**Response options**	1–Yes 2–No	1–Yes 2–No
**Ever belonged to a religious denomination**		
**Question**	C13	Q14
**Question stem**	Have you ever considered yourself as belonging to any particular religion or denomination?	Did you ever belong to a religious denomination?
**Response options**	1–Yes 2–No	1–Yes 2–No
**Target variable**	Dummy coded 0–No 1–Yes	

100% overlapping score

**Table 4:** Attendance at religious services items

	ESS	EVS
**Question #**	C16	Q15
**Question stem**	Apart from special occasions such as weddings and funerals, about how often do you attend religious services nowadays?	Apart from weddings, funerals and christenings, about how often do you attend religious services these days?
**Response options**	1–Every day 2–More than once a week 3–Once a week 4–At least once a month 5–Only on special holy days 6–Less often 7–Never	1–More than once a week 2–Once a week 3–Once a month 4–Only on specific holy days 5–Once a year 6–Less often 7–Never, practically never
**Target variable**	Reverse coded 0–Never 1–Less often (Categories #5 and #6 from the EVS were combined) 2–Only on special holy days 3–Once a month 4–Once a week 5–More than once a week (Categories #1 and #2 from the ESS were combined)	

94% overlapping score. Slight differences in label qualifiers

**Table 5:** Praying frequency items

	ESS	EVS
**Question #**	C17	Q22
**Question stem**	Apart from when you are at religious services, how often, if at all, do you pray? Please use this card.	How often do you pray outside of religious services? Would you say…
**Response options**	1–Every day 2–More than once a week 3–Once a week 4–At least once a month 5–Only on special holy days 6–Less often 7–Never	1–Every day 2–More than once a week 3–Once a week 4–At least once a month 5–Several times a year 6–Less often 7–Never
**Target variable**	Reverse coded 1–Never 2–Less often 3–Only on special holy days/several times a year 4–At least once a month 5–Once a week 6–More than once a week 7–Every day	

88% overlapping score. Category #5 in ESS and EVS differ in their wording

**Table 6:** Descriptive statistics of sociodemographic variables and religiosity by group

	EVS (***N*** = 21513)				ESS (***N*** = 29565)			
	**EVS DE (*****N*****= 1460)**				**ESS DE (*****N*****= 2258)**			
	**Mean**	**SE**	**Min**	**Max**	**Mean**	**SE**	**Min**	**Max**
Age (respondent)	49.61	0.482	18	82	50.21	0.384	18	82
Female	0.512	0.013	0	1	0.512	0.011	0	1
Educational level (respondent)	1.962	0.017	1	3	1.598	0.016	1	3
In paid work	0.564	0.013	0	1	0.572	0.010	0	1
Religious person (joint)	0.539	0.013	0	1	4.222	0.065	0	10
	**EVS SI (*****N*****= 1023)**				**ESS SI (*****N*****= 1263)**			
	**Mean**	**SE**	**Min**	**Max**	**Mean**	**SE**	**Min**	**Max**
Age (respondent)	49.13	0.543	18	82	50.05	0.501	18	82
Female	0.500	0.016	0	1	0.508	0.014	0	1
Educational level (respondent)	1.763	0.024	1	3	1.821	0.021	1	3
In paid work	0.552	0.016	0	1	0.546	0.014	0	1
Religious person (joint)	0.689	0.014	0	1	4.597	0.091	0	10
	**EVS NO (*****N*****= 950)**				**ESS NO (*****N*****= 1312)**			
	**Mean**	**SE**	**Min**	**Max**	**Mean**	**SE**	**Min**	**Max**
Age (respondent)	48.05	0.590	18	82	47.70	0.498	18	82
Female	0.485	0.016	0	1	0.481	0.014	0	1
Educational level (respondent)	1.902	0.027	1	3	1.915	0.024	1	3
In paid work	0.623	0.016	0	1	0.654	0.013	0	1
Religious person (joint)	0.371	0.016	0	1	3.273	0.076	0	10
	**EVS EE (*****N*****= 1260)**				**ESS EE (*****N*****= 1841)**			
	**Mean**	**SE**	**Min**	**Max**	**Mean**	**SE**	**Min**	**Max**
Age (respondent)	49.60	0.517	18	82	49.38	0.424	18	82
Female	0.538	0.014	0	1	0.539	0.012	0	1
Educational level (respondent)	2.124	0.020	1	3	2.181	0.016	1	3
In paid work	0.599	0.014	0	1	0.645	0.011	0	1
Religious person (joint)	0.354	0.014	0	1	3.245	0.072	0	10
	**EVS SE (*****N*****= 1138)**				**ESS SE (*****N*****= 1479)**			
	**Mean**	**SE**	**Min**	**Max**	**Mean**	**SE**	**Min**	**Max**
Age (respondent)	45.39	0.514	18	82	46.78	0.470	18	82
Female	0.488	0.015	0	1	0.494	0.013	0	1
Educational level (respondent)	2.030	0.022	1	3	2.025	0.020	1	3
In paid work	0.646	0.014	0	1	0.648	0.012	0	1
Religious person (joint)	0.270	0.013	0	1	2.875	0.074	0	10
	**EVS PL (*****N*****= 1297)**				**ESS PL (N = 1412)**			
	**Mean**	**SE**	**Min**	**Max**	**Mean**	**SE**	**Min**	**Max**
Age (respondent)	46.66	0.503	18	82	48.62	0.474	18	82
Female	0.532	0.014	0	1	0.522	0.013	0	1
Educational level (respondent)	1.737	0.025	1	3	1.785	0.022	1	3
In paid work	0.544	0.014	0	1	0.562	0.013	0	1
Religious person (joint)	0.858	0.010	0	1	6.113	0.072	0	10
	**EVS NL (*****N*****= 660)**				**ESS NL (*****N*****= 1572)**			
	**Mean**	**SE**	**Min**	**Max**	**Mean**	**SE**	**Min**	**Max**
Age (respondent)	44.66	0.674	18	82	48.82	0.451	18	82
Female	0.439	0.019	0	1	0.510	0.013	0	1
Educational level (respondent)	1.787	0.031	1	3	1.705	0.022	1	3
In paid work	0.621	0.019	0	1	0.613	0.012	0	1
Religious person (joint)	0.491	0.020	0	1	3.988	0.080	0	10
	**EVS CH (*****N*****= 642)**				**ESS CH (*****N*****= 1456)**			
	**Mean**	**SE**	**Min**	**Max**	**Mean**	**SE**	**Min**	**Max**
Age (respondent)	49.01	0.721	18	82	48.73	0.470	18	82
Female	0.503	0.020	0	1	0.508	0.013	0	1
Educational level (respondent)	2.013	0.023	1	3	1.686	0.021	1	3
In paid work	0.619	0.019	0	1	0.629	0.013	0	1
Religious person (joint)	0.573	0.020	0	1	4.756	0.080	0	10
	**EVS FI (*****N*****= 372)**				**ESS FI (*****N*****= 1684)**			
	**Mean**	**SE**	**Min**	**Max**	**Mean**	**SE**	**Min**	**Max**
Age (respondent)	52.56	0.972	18	82	50.41	0.454	18	82
Female	0.465	0.026	0	1	0.511	0.012	0	1
Educational level (respondent)	1.892	0.036	1	3	2.066	0.017	1	3
In paid work	0.523	0.026	0	1	0.542	0.012	0	1
Religious person (joint)	0.595	0.026	0	1	4.789	0.070	0	10
	**EVS GB (*****N*****= 1747)**				**ESS GB (*****N*****= 2097)**			
	**Mean**	**SE**	**Min**	**Max**	**Mean**	**SE**	**Min**	**Max**
Age (respondent)	48.66	0.434	18	82	48.04	0.386	18	82
Female	0.511	0.012	0	1	0.506	0.011	0	1
Educational level (respondent)	1.835	0.020	1	3	1.992	0.018	1	3
In paid work	0.585	0.012	0	1	0.625	0.011	0	1
Religious person (joint)	0.371	0.012	0	1	3.508	0.068	0	10
	**EVS RS (*****N*****= 1369)**				**ESS RS (*****N*****= 1966)**			
	**Mean**	**SE**	**Min**	**Max**	**Mean**	**SE**	**Min**	**Max**
Age (respondent)	49.21	0.479	18	82	46.38	0.390	18	82
Female	0.521	0.014	0	1	0.510	0.011	0	1
Educational level (respondent)	1.409	0.017	1	3	1.746	0.016	1	3
In paid work	0.447	0.013	0	1	0.439	0.011	0	1
Religious person (joint)	0.798	0.011	0	1	5.870	0.068	0	10
	**EVS IT (*****N*****= 2159)**				**ESS IT (*****N*****= 2617)**			
	Mean	SE	Min	Max	Mean	SE	Min	Max
Age (respondent)	51.87	0.387	18	82	51.14	0.361	18	82
Female	0.486	0.011	0	1	0.519	0.010	0	1
Educational level (respondent)	1.540	0.015	1	3	1.578	0.014	1	3
In paid work	0.454	0.011	0	1	0.446	0.010	0	1
Religious person (joint)	0.776	0.001	0	1	5.593	0.055	0	10
	**EVS HU (*****N*****= 1455)**				**ESS HU (*****N*****= 1594)**			
	**Mean**	**SE**	**Min**	**Max**	**Mean**	**SE**	**Min**	**Max**
Age (respondent)	49.25	0.474	19	82	49.55	0.458	18	82
Female	0.529	0.013	0	1	0.527	0.013	0	1
Educational level (respondent)	1.726	0.020	1	3	1.721	0.019	1	3
In paid work	0.569	0.013	0	1	0.596	0.012	0	1
Religious person (joint)	0.549	0.013	0	1	3.734	0.073	0	10
	**EVS FR (*****N*****= 1817)**				**ESS FR (*****N*****= 1941)**			
	**Mean**	**SE**	**Min**	**Max**	**Mean**	**SE**	**Min**	**Max**
Age (respondent)	50.08	0.434	18	82	49.78	0.414	18	82
Female	0.520	0.012	0	1	0.526	0.011	0	1
Educational level (respondent)	1.617	0.019	1	3	1.699	0.018	1	3
In paid work	0.474	0.012	0	1	0.528	0.011	0	1
Religious person (joint)	0.415	0.012	0	1	4.778	0.079	0	10
	**EVS AT (*****N*****= 1560)**				**ESS AT (*****N*****= 2414)**			
	**Mean**	**SE**	**Min**	**Max**	**Mean**	**SE**	**Min**	**Max**
Age (respondent)	47.93	0.469	18	82	49.46	0.359	18	82
Female	0.513	0.013	0	1	0.519	0.010	0	1
Educational level (respondent)	2.031	0.017	1	3	1.665	0.017	1	3
In paid work	0.610	0.012	0	1	0.574	0.010	0	1
Religious person (joint)	0.609	0.012	0	1	4.804	0.062	0	10
	**EVS DK (*****N*****= 1671)**				**ESS DK (*****N*****= 1501)**			
	**Mean**	**SE**	**Min**	**Max**	**Mean**	**SE**	**Min**	**Max**
Age (respondent)	41.13	0.402	18	82	49.53	0.477	18	82
Female	0.479	0.012	0	1	0.505	0.013	0	1
Educational level (respondent)	1.979	0.018	1	3	1.693	0.022	1	3
In paid work	0.698	0.011	0	1	0.583	0.013	0	1
Religious person (joint)	0.534	0.012	0	1	3.935	0.072	0	10
	**EVS ME (*****N*****= 933)**				**ESS ME (*****n*****= 1158)**			
	**Mean**	**SE**	**Min**	**Max**	**Mean**	**SE**	**Min**	**Max**
Age (respondent)	46.07	0.572	18	82	45.00	0.489	18	82
Female	0.513	0.016	0	1	0.511	0.015	0	1
Educational level (respondent)	1.711	0.021	1	3	1.939	0.020	1	3
In paid work	0.411	0.016	0	1	0.444	0.015	0	1
Religious person (joint)	0.885	0.011	0	1	5.180	0.084	0	10

Source: EVS 5(2020a), ESS 9(2018)-weighted

*SE* standard error, *min* minimum, *max* maximum

**Table 7:** Characteristics of the sociodemographic variables and their use in the analysis

***Variable***	***ESS R9***	***EVS W5***	***Target***
*Age*	*agea* Numeric	*age* Numeric	*hage* Numeric (EVS data top coded at 82+ for consistency with ESS data)
*Sex*	*gndr* 1 Male 2 Female	*v225* 1 Male 2 Female	*hfemale* 0 Male 1 Female
*Education level*	*eisced* 1 Less than lower secondary 2 Lower secondary 3 Lower tier upper secondary 4 Upper tier upper secondary 5 Advanced vocational 6 Lower tertiary 7 Higher tertiary 55 Other	*v243_EISCED* 0 No formal education 1 Primary 2 Lower secondary 3 Lower tier upper secondary 4 Upper tier upper secondary 5 Advanced vocational 6 Bachelor’s level 7 Master’s and higher 6666 Other	*edu* 1 Lower secondary and below (1–3 ESS; 0–3 EVS) 2 Upper secondary/non-tertiary (4–5) 3 Tertiary (6–7) (Other recoded to “no answer”)
*Paid work*	*Pdwrkn* (paid work, last 7 days) 0 Marked 1 Marked	*v244 (paid employment)* 1 30 h a week or more 2 Less than 30 h a week 3 Self-employed 4 Military service 5 Retired 6 Homemaker 7 Student 8 Unemployed 9 Disabled 10 Other	*hpaidwrk* 0 Not in paid work 1 In paid work (1–3)
*Individual religiosity*	*C15* 0–Not at all religious 1 2 3 4 5 6 7 8 9 10–Very religious	*Q17* 1–A religious person 2–Not a religious person 3–A convinced atheist	*jreligious* *EVS:* 0–Not religious/atheist (Categories #2 and #3) 1–Religious *ESS:* 0 (Not at all religious)–10 (Very religious)

**Table 8:** Steps adopted to maximise comparability between the ESS and EVS data

***Differences***	***Harmonisation***		
**Mode of data collection**	Exclusion of the self-administered and telephone surveys conducted in Wave 5 of the EVS, limiting the comparisons to face-to-face, full length interviews. New variable created to specify survey mode (CAPI, PAPI)		
**Study population**	Included sample	EVS	ESS
	Exclusion of respondents aged 15, 16, and 17 from the ESS data, so that both samples include individuals 18 and over	Target population 18+	Target population starting from 15 years old
**Non-substantive responses**	Harmonise missing data codes, differentiating among:	EVS	ESS
	- “Don’t know” - “No answer” - “Refusal”	−1 −5, −8, −9, −10 −2	.c .d .b

*CAPI* computer-assisted personal interview, *PAPI* pen-and-paper personal interview

## Appendix 2

Figures 5, 6 and 7

Table 9

**Table 9:** Proportion tests of non-substantive responses for items, country and survey

	DE	SI	NO	EE	SE	PL	NL	CH	FI	GB	RS	IT	HU	FR	AT	DK	ME
**Current religious denomination**																	
Proportion (EVS)	0	.605	0	.226	.277	.987	.655	.191	0	0	1.115	2.632	1.588	.264	.362	.335	1.214
Proportion (ESS)	.214	.498	.868	0	.248	2.040	.189	.352	.385	.118	.919	1.458	1.779	.410	.246	.278	.779
Diff (Prop.)	−.214	.107	−.868	.226	.029	−1.054	.466	−.161	−.385	−.118	.195	1.173	−.190	−.146	.116	.057	.435
z	−1.791	.351	−2.920	2.043	.145	−2.267	1.784	−.632	−1.224	−1.455	.574	2.944	−.414	−.776	.675	.291	1.029
*p*-value	.073	.726	.004	.041	.885	.023	.074	.527	.221	.146	.566	.003	.679	.438	.499	.771	.304
**Past religious denomination**																	
Proportion (EVS)	0.000	0.016	0.000	0.002	0.012	0.033	0.011	0.015	0.000	0.001	0.049	0.076	0.015	0.001	0.002	0.020	0.103
Proportion (ESS)	0.001	0.017	0.007	0.000	0.004	0.092	0.001	0.012	0.000	0.000	0.036	0.066	0.032	0.006	0.011	0.006	0.043
Diff. (prop.)	−0.001	−0.001	−0.007	0.002	0.007	−0.059	0.010	0.004	NA	0.001	0.013	0.010	−0.017	−0.005	−0.009	0.014	0.060
z	−0.771	−0.136	−1.574	1.671	1.506	−1.998	3.013	0.394	NA	0.690	0.936	0.679	−2.242	−1.835	−1.730	1.916	2.526
*p*-value	.441	.892	.115	.095	.132	.046	.003	.693	NA	.490	.349	.497	.025	.067	.084	.055	.012
**Attendance at religious services**																	
Proportion (EVS)	0.055	0.462	0.080	0.320	0.103	1.141	0.377	0.739	0.218	0	2.088	1.946	0.783	0.200	0.358	0.216	1.627
Proportion (ESS)	0.343	0.500	0.099	0.041	0.476	2.745	0.045	0.209	0.214	0.041	0.863	1.557	1.652	0.290	0.493	0.132	0.875
Diff. (prop.)	−.287	−.038	−.019	.279	−.004	−.016	.332	.529	.004	−.041	1.226	.389	−.869	−.090	−.136	.084	.752
z	−1.806	−.132	−.151	1.949	−1.727	−3.049	1.911	1.866	.016	−.860	3.073	1.045	−2.205	−.564	−.644	.563	1.595
*p*-value	.071	.895	.880	.051	.084	.002	.056	.062	.987	.390	.002	.296	.027	.573	.520	.574	.111
**Praying frequency**																	
Proportion (EVS)	0.595	1.162	0.109	2.681	0.035	2.355	0.355	1.046	0.727	0.324	2.895	2.999	2.234	0.528	2.365	0.216	3.047
Proportion (ESS)	1.022	1.102	0.341	0.292	0.295	8.377	0.472	1.028	0.184	0.228	2.789	3.825	4.265	0.940	3.546	0.563	1.296
Diff. (prop.)	−.427	.061	−.232	2.389	−.260	−6.022	−.117	.018	.543	.096	.105	−.826	−2.031	−.413	−1.181	−.347	1.751
z	−1.395	.138	−1.118	5.874	−1.583	−7.001	−.389	.037	1.810	.576	.186	−1.590	−3.192	−1.492	−2.147	−1.596	2.843
*p*-value	.163	.889	.263	.000	.113	.000	.697	.970	.070	.564	.852	.112	.001	.136	.032	.110	.004

*Source:* EVS 5(2020a), ESS 9(2018) – weighted

Figure 8

## Footnotes

1 With the difference that ESS targets the resident population from 15 years old, EVS from 18 years old.

2 Computer-assisted personal interviewing (CAPI) is used for ESS Round 9 and self-administered mode was allowed during the fieldwork of the latest ESS Round 10 due to challenges presented by the COVID-19 pandemic, with some countries collecting data using face-to-face interviews, and others self-administered modes (web and mail).

3 The ESS-SUSTAIN-2–European Social Survey Sustainability–is a €4.9m joint project (grant agreement number 676166) coordinated by the European Social Survey together with other 16 partner countries/research institutes across Europe. It aims at improving research methodology and analytical power of collected data, in a more cost-effective manner, and to achieve long-term sustainability. One focus point of SUSTAIN-2 is the exploration of bridging the survey infrastructures of the EVS and the ESS. Questionnaire and item comparison undertaken and reported in this paper will possibly allow EVS items to be collected using the ESS infrastructure. For more information see https://www.europeansocialsurvey.org/about/funding.html.

4 ESS Round 9 was preferred over ESS Round 10 due to the period of data collection being closer to that of EVS Wave 5.

5 This preliminary analytical step yielded a list of 24 substantive items with a potential conceptual overlap across the two surveys.

6 Except for the Mann-Whitney U test, which did not support the use of weighted data.

7 Post-stratification weight and calibration weights were respectively used in the ESS and the EVS (design weights for Germany). More information about the specific weights is provided in the references (EVS, 2020a; Kaminska, 2020).

8 By sampling strategy we refer to using a similar sample frame so that the same register of individuals across the countries could be used for comparison. This way differences attributed to coverage errors (Ortmanns, 2020) and design effects (design weights are only available for five EVS countries) are minimised.
