# Supplementary material for: Exploring the feasibility of ex-post harmonisation of religiosity items from the European Social Survey and the European Values Study
Source: Meas Instrum Soc Sci. 2022 Sep 30;4(1):12. doi: 10.1186/s42409-022-00038-x (PMC9523191; doi:10.1186/s42409-022-00038-x)
Supplement: Supplementary file 1 — Additional file 1: Table S1. Comparison of EVS Wave 5 and ESS Round 10 items for belonging to a religious denomination. [file 42409_2022_38_MOESM1_ESM.docx]

Table S1 *Comparison of EVS Wave 5 and ESS Round 10 items for belonging to a religious denomination*

|  | **EVS W5** | **ESS R10** |
| --- | --- | --- |
| **Question wording** | Do you belong to a religious denomination?  *Yes - No* | Do you consider yourself as belonging to any particular religion or denomination?  *Yes - No* |
| **Question attributes** | | |
| Reference period | Present | Present |
| Ref. period details | NA | NA |
| Balance of the request | Not applicable | Not applicable |
| Part of a battery | No | No |
| Contingent on filter | No | Yes |
| **Interviewer role** | | |
| Clarifications | Not present | Not present |
| Instructions | None | None |
| **Response attributes** | | |
| Variable type | Dichotomous | Dichotomous |
| Number of categories | 2 | 2 |
| Range of values | 1;2 | 1;2 |
| Labels | Full | Full |
| Label order | Decremental | Decremental |
| Polarity | NA | NA |
| Neutral category | NA | NA |
| Scale symmetry | NA | NA |
| **Showcards** |  |  |
| Showcards | None | None |
| Layout | NA | NA |
| **Overlapping score** | 100 | |
