# Supplementary material for: Exploring the feasibility of ex-post harmonisation of religiosity items from the European Social Survey and the European Values Study
Source: Meas Instrum Soc Sci. 2022 Sep 30;4(1):12. doi: 10.1186/s42409-022-00038-x (PMC9523191; doi:10.1186/s42409-022-00038-x)
Supplement: Supplementary file 2 — Additional file 2: Table S2. Comparison of EVS Wave 5 and ESS Round 10 items for having belonged to a religious denomination. [file 42409_2022_38_MOESM2_ESM.docx]

Table S2 *Comparison of EVS Wave 5 and ESS Round 10 items for having belonged to a religious denomination*

|  | **EVS W5** | **ESS R10** |
| --- | --- | --- |
| **Question wording** | Did you ever belong to a religious denomination?  *Yes - No* | Have you ever considered yourself as belonging to any particular religion or denomination?  *Yes - No* |
| **Question attributes** | | |
| Reference period | Past | Past |
| Ref. period details | Ever | Ever |
| Balance of the request | Not applicable | Not Applicable |
| Part of a battery | No | No |
| Contingent on filter | Yes | Yes |
| **Interviewer role** | | |
| Clarifications | Not present | Not present |
| Instructions | None | None |
| **Response attributes** | | |
| Variable type | Dichotomous | Dichotomous |
| Number of categories | 2 | 2 |
| Range of values | 1;2 | 1;2 |
| Labels | Full | Full |
| Label order | Decremental | Decremental |
| Polarity | NA | NA |
| Neutral category | NA | NA |
| Scale symmetry | NA | NA |
| **Showcards** |  |  |
| Showcards | None | None |
| Layout | NA | NA |
| **Overlapping score** | 100 | |
