# Supplementary material for: Exploring the feasibility of ex-post harmonisation of religiosity items from the European Social Survey and the European Values Study
Source: Meas Instrum Soc Sci. 2022 Sep 30;4(1):12. doi: 10.1186/s42409-022-00038-x (PMC9523191; doi:10.1186/s42409-022-00038-x)
Supplement: Supplementary file 4 — Additional file 4: Table S4. Comparison of EVS Wave 5 and ESS Round 10 items for praying frequency. [file 42409_2022_38_MOESM4_ESM.docx]

Table S4 *Comparison of EVS Wave 5 and ESS Round 10 items for praying frequency*

|  | **EVS W5** | **ESS R10** |
| --- | --- | --- |
| **Question wording** | How often do you pray outside of religious services? Would you say … *Every day – never* | Apart from when you are at religious services, how often, if at all, do you pray? Please use this card.  *Every day – never* |
| **Question attributes** | | |
| Reference period | Present | Present |
| Ref. period details | Outside of religious services | Outside of religious services |
| Balance of the request | Not applicable | Balanced |
| Part of a battery | No | No |
| Contingent on filter | No | No |
| **Interviewer role** | | |
| Clarifications | Not present | Not present |
| Instructions | Interviewer | None |
| **Response attributes** | | |
| Variable type | Ordinal | Ordinal |
| Number of categories | 7 | 7 |
| Range of values | 1-7 | 1-7 |
| Labels | Fully labelled | Fully labelled |
| Label order | Decremental | Decremental |
| Polarity | Unipolar | Unipolar |
| Neutral category | Not applicable | Not applicable |
| Scale symmetry | Not applicable | Not applicable |
| **Showcards** |  |  |
| Showcards | Response scale | Response scale |
| Layout | Vertical | Vertical |
| **Overlapping score** | 88 | |
